# Risk-stratified surveillance for hepatocellular carcinoma

**DOI:** 10.1097/HC9.0000000000000943

**Published:** 2026-03-31

**Authors:** Thomas Hunold, Neehar D. Parikh

**Affiliations:** Division of Gastroenterology and Hepatology, University of Michigan, Ann Arbor, Michigan, USA

**Keywords:** cirrhosis, hepatocellular carcinoma, hepatitis B, implementation, screening

## Abstract

Hepatocellular carcinoma (HCC) is a leading cause of cancer-related mortality worldwide, with most cases arising in patients with cirrhosis or chronic hepatitis B. Current guidelines recommend semi-annual ultrasound with or without alpha-fetoprotein (AFP) testing for all at-risk individuals; however, this one-size-fits-all approach has important limitations, including suboptimal sensitivity, frequent false-positive results, poor adherence, and failure to account for substantial heterogeneity in individual HCC risk. Risk-stratified surveillance has emerged as a potential strategy to better balance surveillance benefits and harms by tailoring surveillance intensity and modality to patient-specific risk. We summarize and critically evaluate existing HCC risk stratification models, including clinical, biomarker-based, and imaging-based or elastography-based approaches. Although several models demonstrate promising performance, many lack robust external validation, underrepresent patients with metabolic and alcohol-associated liver disease, and inadequately account for competing risks such as non–liver-related mortality. We further discuss key challenges to implementing risk-stratified surveillance in clinical practice. Overall, while risk-stratified HCC surveillance has several promising characteristics over current paradigms, prospective validation and implementation studies are needed before widespread adoption. Aligning surveillance strategies with individualized risk has the potential to improve outcomes in patients at risk for HCC.

## INTRODUCTION

Hepatocellular carcinoma (HCC) is a leading cause of cancer-related morbidity and mortality worldwide.^1^,^2^ Early detection is associated with improved outcomes through the application of curative therapies (ie, surgery, ablation, transplantation), resulting in 5-year survival rates that can exceed 70%.^3^,^4^

The vast majority of HCCs develop in at-risk patients with underlying cirrhosis or chronic hepatitis B. The incidence of HCC in these at-risk groups varies significantly depending on the etiology of liver disease and patient-level factors: ~1%–2% annually in patients with cirrhosis from steatotic liver diseases [alcohol-associated liver disease (ALD) and metabolic dysfunction–associated steatotic liver disease (MASLD)] and cured viral hepatitis, while annual risk can be as high as 4%–6% in patients with cirrhosis from viral hepatitis.^5^,^6^,^7^,^8^ Demographic and behavioral factors associated with increased incidence of HCC include male sex, tobacco use, alcohol intake, and obesity.^8^,^9^,^10^,^11^,^12^

Guidelines from the American Association for the Study of Liver Diseases (AASLD), European Association for the Study of the Liver (EASL), and the Asian Pacific Association for the Study of the Liver (APASL) currently recommend a “one-size-fits-all” surveillance approach for HCC in at-risk patients with ultrasound with or without alpha-fetoprotein (AFP) testing every 6 months.^13^,^14^,^15^ The guideline recommendations are based on evidence from meta-analyses of cohort studies (level II evidence) showing that HCC surveillance is associated with improved survival, early stage detection, and receipt of curative therapy.^4^ Modeling studies suggest that the threshold HCC incidence for cost-effective surveillance with ultrasound and AFP is ~0.4%, supporting surveillance even in relatively low-risk individuals.^16^,^17^ Notably, however, we lack level I evidence from randomized controlled trials of the benefits of HCC surveillance, which has prevented its adoption into national screening guidelines from the US Preventive Services Task Force. Nevertheless, given the broad range of HCC risk for which surveillance with ultrasound and AFP is cost-effective, universal screening in at-risk patients is currently recommended by societal guidelines.

Risk-stratified screening paradigms have been instituted in other cancer types, including colon cancer screening and breast cancer screening. In colon cancer, given the influence of germline mutations on risk, family history plays a role in the age of initiation of colon cancer screening and the surveillance interval.^18^ In breast cancer screening, there are polygenic risk scores that have been developed for breast cancer risk, and one was recently evaluated in a risk-stratified screening trial. Use of the score for tailoring screening intensity and modality showed noninferiority to usual care in detecting advanced-stage cancers with broad acceptance risk-based screening in the vast majority of patients enrolled in the trial.^19^ Surveillance for HCC is unique as polygenic risk scores have not been shown to add any discrimination in risk, as the majority of risk can be traced to somatic factors, including demographics, severity of liver disease, and behavioral factors, which makes any assessment of risk more complex.^20^ However, effective risk stratification may mitigate some of the limitations of current HCC surveillance programs.

Ultrasound and AFP have limitations in sensitivity, specificity, and poor adherence, which collectively limit the effectiveness of HCC surveillance. Surveillance with ultrasound and AFP misses up to one-third of early-stage HCCs, with diminished sensitivity in patients with poor visualization on ultrasound.^16^ False positives are frequent in patients undergoing surveillance, leading to harms such as excessive imaging and psychological distress.^21^,^22^ Finally, poor adherence substantially limits the effectiveness of imaging-based surveillance, with a meta-analysis reporting surveillance rates below 10% among at-risk patients.^17^ Poor adherence is a result of the intense nature of HCC surveillance, with semi-annual testing, patient/provider barriers to test completion and ordering, and lack of level I evidence supporting the benefits of HCC surveillance.^23^,^24^,^25^

**FIGURE 1:**
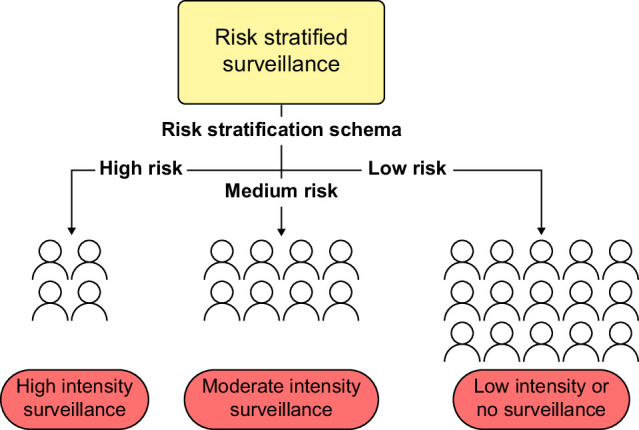
Potential schema of risk-based HCC surveillance where high-risk individuals undergo high-intensity surveillance, such as abdominal MRI-based surveillance, moderate-risk individuals undergo standard intensity surveillance, such as semi-annual abdominal ultrasound and alpha-fetoprotein, and low-risk individuals could potentially forgo surveillance. Abbreviation: HCC, hepatocellular carcinoma.

HCC surveillance strategies based on risk stratification may mitigate several limitations of current approaches. Risk stratification can maximize the benefits of HCC surveillance in higher-risk individuals while minimizing harms in lower-risk individuals (Figure 1). For example, efforts to improve adherence could be targeted toward high-risk individuals, or such individuals could undergo surveillance with more sensitive methods, such as CT/MRI or abbreviated MRI.^26^,^27^ Further benefits of risk stratification include targeted efforts to improve risk in high-risk individuals through behavioral change or chemoprevention.^28^ Risk-based surveillance has been endorsed by the European Association for the Study of the Liver; however, its clinical effectiveness and feasibility of implementation have not yet been established.^29^ Although several risk stratification models have been described, few have undergone robust validation or been implemented in routine clinical practice. Here, we review clinical risk stratification models and discuss challenges related to their implementation.

## CLINICAL RISK STRATIFICATION MODELS

### aMAP score

The aMAP score combines age, gender, albumin-bilirubin (ALBI) score, and platelet count with a scaled score from 0 to 100. The score was derived from a cohort of 3688 patients with chronic hepatitis B and validated in mixed international cohorts of patients with hepatitis C and nonviral hepatitis (n=13,686). The aMAP score showed good performance in patients with viral hepatitis (c-statistic 0.82), and the validation cohorts had similar levels of performance (c-statistic 0.82–0.87).^30^ The 5-year cumulative incidences of HCC were 0.8%, 4.8%, and 17.8% in the low-risk, medium-risk, and high-risk groups, respectively. Subsequent validation in mixed at-risk populations demonstrated similar performance (c-statistics 0.81–0.83).^30^,^31^,^32^

### Toronto HCC risk index

The THRI combines age, gender, etiology of liver disease, and platelet count and stratifies risk into low-risk, medium-risk, and high-risk groups. The score was derived from a single-center cohort of 2079 patients with cirrhosis, of whom 226 developed HCC over a median of 5.4 years. The 10-year cumulative HCC incidence was 3%, 10%, and 32% in the low-risk, medium-risk, and high-risk groups, respectively. The model has been externally validated in a cohort of 1144 patients with primary biliary cholangitis and viral etiologies of cirrhosis with good performance (c-statistic 0.77).^33^ The THRI has subsequently been validated in an additional cohort of 2491 patients, nearly half of whom had metabolic causes of liver disease and had diminished performance (c-statistic 0.69) with only a small proportion of low-risk patients identified in the cohort (5.3%), potentially limiting clinical utility.^34^

### ADRESS-HCC

The ADRESS-HCC model includes age, race, diabetes, etiology, gender, Child–Pugh score, and was derived from 34,932 patients waitlisted for liver transplantation in the United States, of which 1960 patients developed HCC over a median follow-up of 1.3 years. The c-statistic for 1-year HCC risk in the cohort was 0.704 with external validation in the Hepatitis C Antiviral Long-term Treatment against Cirrhosis (HALT-C) cohort (n=426; 29 with incident HCC over a 5-year follow-up), showing a similar performance (c-statistic 0.691).^35^ The range of annual HCC incidence ranged from 0.2% to 4.6% using the model.^35^ Further validation in more contemporary populations of patients has yet to be conducted.

### HCC risk score

The HCC risk score was derived and internally validated in a cohort of 23,234 patients with metabolic causes of liver disease. There were 1278 incident HCCs over a median follow-up of 3.7 years. The model consists of age, gender, diabetes, body mass index, platelet count, serum albumin, and the aspartate aminotransferase-to-alanine aminotransferase ratio. In internal cross-validation, the c-statistic was 0.75–0.76 in cohorts with MASLD and ALD cirrhosis, respectively.^36^ The HCC risk score has yet to be externally validated.

### Alcohol-associated liver cancer estimation score

The alcohol-associated liver cancer estimation (ALICE) score was derived and validated in a single-center cohort of 745 Korean patients with alcohol-associated cirrhosis, with the cohort split into derivation (n=507) and validation (n=238) cohorts. Over a median follow-up of 59.0 months, 62 patients developed incident HCC. The derived score includes age, albumin, and AFP levels to calculate a nomogram-based score with 3 risk strata: low risk (≤60), intermediate risk (60–100), and high risk >100. The investigators accounted for competing risks of death and liver transplantation in the modeling approach. The score demonstrated good discrimination, with an AUC of 0.796 for 12-month HCC risk in the validation cohort. Estimates of 5-year risk prediction were slightly lower at 0.758.^37^ Clinical applicability is limited by the absence of external validation in more diverse populations.

### HCC Risk Stratification Index

The HCC Risk Stratification Index was derived from a combination of 2 prospective cohorts (n=2431), the Texas Hepatocellular Carcinoma Consortium Cohort and the Houston Veterans Administration Cirrhosis Surveillance Cohort.^38^ The derivation cohort had 119 incident HCCs over a median follow-up of 3.2 years. The derived model included age, gender, smoking, alcohol use, body mass index, etiology, AFP, albumin, alanine aminotransferase, and platelet levels. The model showed good performance on internal validation with a c-statistic of 0.75 for the 1-year risk of HCC. The model was validated in an external cohort of 20,728 US Veterans with cirrhosis, of whom 898 developed HCC over a median of 2.4 years and had a lower c-statistic of 0.70.^38^ While informative, this level of discrimination is likely insufficient for routine clinical implementation, and further validation in non-VA populations is warranted.

### Nahon et al.

The HCC risk stratification score developed by Nahon et al. incorporates age, gender, platelet count, gamma-glutamyl transferase, bilirubin, and AFP, yielding a score ranging from 0 to 9. The model was derived from a multinational cohort of 1658 patients with compensated cirrhosis, among whom 131 developed HCC over a median follow-up of 37.0 months. In the derivation cohort, the c-index for 5-year HCC risk prediction was 0.737, with approximately one-third of patients classified as high risk (annual incidence 5.5%). External validation in a cohort of 855 patients with compensated cirrhosis and 75 incident HCC cases over a median follow-up of 37.2 months demonstrated similar discrimination (c-index 0.723), with an annual HCC incidence of 5.36% in the high-risk group.^39^ This score was specifically designed to identify patients suitable for MRI-based surveillance and is currently being used as an inclusion criterion for the prospective FASTTRAK trial, which is evaluating abbreviated MRI surveillance in patients with an annual HCC incidence >3.0%.^40^

### PAGE-B score

The PAGE-B score consists of age, gender, and platelet count to stratify the risk of HCC in patients with chronic hepatitis B on antiviral therapy. The score was derived from a multicenter cohort of 1325 patients with a 5-year incidence of HCC of 5.7%. The c-index in the derivation set was 0.82 with a stratified 5-year incidence of 0%, 3%, and 17% in the low, medium, and high-risk groups, respectively. The score was subsequently validated in a single-center cohort of 490 patients with a 5-year HCC incidence of 8.7%. The c-index was also 0.82 with a similar stratified 5-year incidence of 0%, 4%, and 16%.^41^ PAGE-B has been incorporated into professional society guidelines for HCC surveillance, given its consistent validation across cohorts.^13^ Modifications of the PAGE-B, including inclusion of albumin, may improve risk stratification in certain populations, although further validation is required.^42^

### REACH-B score

The REACH-B score consists of age, gender, alanine aminotransferase, hepatitis B e antigen status, and hepatitis B DNA and was derived from a non-cirrhotic cohort of 3584 patients with chronic hepatitis B not receiving antiviral therapy. Over a median follow-up of 12.0 years, 131 patients developed incident HCC. The score was externally validated in a cohort of 1505 patients with similar characteristics, among whom 111 developed HCC over a median follow-up of 7.3 years. REACH-B stratifies patients into 17 risk categories, with 5-year HCC risk estimates ranging from 0% to 47.4% (c-index 0.796).^43^ Due to its complexity and limited applicability to contemporary populations, uptake of REACH-B in clinical practice has been modest.

Of the clinical models for HCC risk stratification, few, except for PAGE-B, have been integrated into clinical society guidelines for use in clinical practice. This is in part due to either a lack of adequate validation or a lack of demonstration of effectiveness in population-based surveillance programs. The aMAP score has undergone the most extensive testing in retrospective cohorts, but has not been prospectively validated in clinical practice. The most common factors included in the models include age, gender, and markers of liver function or portal hypertension. While these variables are clinically available and routinely measured, they may decrease their performance in risk stratification when competing risks are considered (Table 1).

**TABLE 1:** Select clinical models for hepatocellular carcinoma risk stratification

Risk stratification study	Components	Derivation	Validation
Fan, 2020—aMAP score^30^	Age, gender, albumin-bilirubin score, and platelet count	3688 patients with chronic hepatitis B, with 95 developing HCC over a median follow-up of 42.7 mo; c-statistic 0.82	13,686 patients with mixed etiologies of chronic liver disease with 3-year or 5-year incidence of HCC ranging from 1.3% to7.0%; c-statistic 0.82–0.87
Ioannou, 2019— HCC risk score^36^	Age, gender, diabetes, BMI, platelet count, serum albumin, and aspartate aminotransferase to alanine aminotransferase ratio	23,234 patients with NASH or alcohol-associated cirrhosis; 1237 developed HCC over a median follow-up of 3.7 y	Internal cross-validation: c-statistic: 0.75–0.76
Sharma, 2019— Toronto Hepatocellular Carcinoma Risk Index^33^	Age, gender, etiology, platelet count	2079 patients with cirrhosis, 226 developed HCC over a median 5.4 y; c-statistic: 0.76	1144 patients with cirrhosis, 107 developed HCC over a median of 7.3 y; c-statistic: 0.77
Flemming, 2014— ADRESS-HCC^35^	Age, race, diabetes, etiology, gender, Child–Pugh score	34,392 patients with cirrhosis, 1960 developed HCC over a median follow-up of 1.3 y; c-statistic: 0.704	426 patients with cirrhosis, 29 developed HCC over a median follow-up of 5.0 y; c-statistic: not reported
Lee, 2022—ALICE score^37^	Age, albumin, AFP	507 patients with alcohol-associated cirrhosis with 62 incident HCCs over a 59 mo median follow-up	238 patients with alcohol-associated cirrhosis with 5-year HCC incidence 6.1%; c-statistic: 0.796 at 12 mo
Kanwal, 2023— HCC Risk Stratification Index^38^	Age, gender, smoking, alcohol use, body mass index, etiology, α-fetoprotein, albumin, alanine aminotransferase, and platelet levels	2431 patients from the VA system with mixed etiologies of cirrhosis with 119 incident HCCs over a median follow-up of 3.2 y; c-statistic: 0.77	20,728 patients with cirrhosis with 898 incident HCCs over a median follow-up of 2.4 y; c-statistic: 0.70
Nahon, 2022^39^	Age, gender, platelet count, GGT level, bilirubin, AFP	1658 patients with cirrhosis (mixed etiology) with 131 incident HCCs over a median follow-up of 37.0 mo; c-statistic: 0.737 for 5-year HCC risk	855 patients with cirrhosis (mixed etiologies) with 75 incident HCCs over a median follow-up of 37.2 mo; c-statistic: 0.723 for 5-year HCC risk
Papatheodoridis, 2016—PAGE-B score^41^	Age, gender, platelet count	1325 patients with chronic hepatitis B on entecavir/tenofovir, 51 developed HCC over a median follow-up of 50 mo; c-statistic: 0.82	490 patients with chronic hepatitis B on entecavir/tenofovir, 34 developed HCC over a median follow-up of 50 mo; c-statistic: 0.82
Yang, 2011— REACH-B score^35^	Age, gender, alanine aminotransferase, hepatitis B e antigen status, hepatitis B DNA	3584 patients with hepatitis B, 131 developed HCC over a median follow-up of 12.0 y	1505 patients with hepatitis B, 111 developed HCC over a median follow-up of 7.3 y; c-statistic (10-year risk): 0.77

Abbreviations: AFP, alpha-fetoprotein; ALICE, alcohol-associated liver cancer estimation; BMI, body mass index; GGT, gamma-glutamyl transferase; HCC, hepatocellular carcinoma; NASH, nonalcoholic steatohepatitis.

## BLOOD BIOMARKER-BASED MODELS

### PLSec score

The PLSec score is a tissue-derived transcriptomic prognostic signature that was developed and validated to predict the HCC risk in at-risk patients. The PLSec-AFP score was derived in a cohort of 331 patients with cirrhosis, among whom 13.9% developed HCC over a median follow-up of 3.5 years. Subsequent validation in 2 nested case-control cohorts with HCV cirrhosis post-SVR (41 HCC and 123 controls; 65 cases and 146 controls) showed good performance in predicting HCC risk (aHR range for both cohorts: 3.08–3.80; 95% CI: 1.66–8.66) with a c-index of 0.74. However, the overall incidence of HCC was relatively high in these cohorts, and prospective validation in contemporary populations—particularly those with steatotic liver disease—is lacking.^44^

### PAAM score

The PAAM score integrates PLSec-AFP with the aMAP score for HCC risk stratification. In the derivation cohort of 331 patients with cirrhosis (13.9% HCC incidence over a median of 4.5 y), the score subsequently underwent blinded validation in the Texas Hepatocellular Carcinoma Consortium (THCCC; n=2156) and the Hepatocellular Carcinoma Early Detection Study (HEDS; n=1328), both phase III biomarker validation cohorts. Validation of the score in the cohorts included death and liver transplantation as competing risks. In the THCCC cohort, the PAAM score stratified patients into high-risk (5.9% annual incidence), intermediate-risk (3.1% annual incidence), and low-risk (0.5% annual incidence) groups. Similar performance was observed in the HEDS cohort, with annual incidences of 5.3%, 2.7%, and 0.6% in the high-risk, intermediate-risk, and low-risk groups, respectively.^45^

Blood-based biomarkers for risk stratification have limited validation, given the need for testing of prospectively collected samples in patients undergoing surveillance. The PAAM score integrates blood-based biomarkers and clinical variables and is the most well-validated in phase 3 cohorts. Despite the favorable performance characteristic, prospective trials evaluating PAAM are needed before clinical implementation.

## IMAGING/LIVER STIFFNESS-BASED MODELS

### VFMAP score

The VFMAP score incorporates transverse shear wave velocity, glucose, sex, age, and AFP. It was derived from a cohort of 1808 patients with chronic liver disease and demonstrated a c-statistic of 0.82 for 5-year HCC risk. A low score (<3) was associated with a 98.2% negative predictive value for HCC development within 5 years.^46^ Subsequent validation in a cohort of 358 patients with a history of hepatitis C infection with sustained virological response (SVR) and a 5.3% incidence of HCC over a median follow-up of 3.2 years, VFMAP was able to stratify the population into low risk (0.96% incidence) and high risk (18.5%) of HCC.^47^

### Semmler et al.

The risk model from Semmler et al.^48^ was developed in a multicenter European cohort of 475 patients with a history of chronic hepatitis C and F3/4 fibrosis after SVR with direct-acting antiviral therapy and compensated liver disease. The model includes AFP, age, liver stiffness measurement, albumin, and alcohol consumption (optional variable). Over a median follow-up of 41 months, the cumulative incidence of HCC was 4.6%. The model stratified 4-year HCC risk from 0.5% in the low-risk group to 16.7% in the high-risk group, with similar discrimination when AFP was excluded. External validation demonstrated reduced ability to identify a truly low-risk group (4-year incidence 3.3%) but preserved identification of high-risk patients (4-year incidence 17.6%).^48^

### PLEASE algorithm

The PLEASE (PLatelet, Elastography, Age, Sex, and Etiologies) algorithm was derived from a multicenter cohort of 1974 patients with advanced chronic liver disease (F3/4 fibrosis) and a liver stiffness measurement by shear wave elastography.^49^ Over a median follow-up of 2.3 years, 106 patients developed HCC. The model includes liver stiffness measurement, age, sex, etiology of liver disease, and platelet count. The c-index on internal validation was 0.874, and the stratified 2-year risk of HCC in the high-risk group was 15.6% (95% CI: 12.1%–18.7%) compared with 1.7% (95% CI: 0.9%–2.5%) in the low-risk group. Two small external validation cohorts showed promising discrimination of HCC-risk based on the PLEASE score, but C-indices were not reported.^49^ Despite its ability to identify high-risk patients, residual HCC risk among low-risk individuals remains substantial, limiting opportunities for meaningful surveillance de-escalation.

The elastography-based models are limited by the validation cohorts lacking an adequate contemporary mix of patients or the ability to stratify patients into both high-risk and low-risk groups. Liver stiffness by elastography is a known risk factor for HCC development; however, the cutoffs for risk stratification in mixed populations of patients with steatotic liver disease are not well established^50^ (Table 2).

**TABLE 2:** Biomarker and imaging/elastography-based models for hepatocellular carcinoma risk stratification

Risk stratification study	Components	Derivation	Validation
Fujiwara, 2021—PLSec-AFP^44^	Transcriptome-based prognostic signature combined with AFP	331 patients with cirrhosis, with 44 patients developing HCC over a median follow-up of 3.5 y	Two nested case-control cohorts of patients with HCV cirrhosis post-SVR (n=41 with HCC/123 with cirrhosis and 65 with HCC/146 with cirrhosis); aHR: 3.80 (95% CI, 1.66–8.66) and aHR: 3.08 (95% CI, 1.78–5.31); c-index 0.74
Fujiwara, 2025—PAAM Score^45^	PLSec-AFP and age, male gender, albumin-bilirubin, and platelets	331 patients with cirrhosis, with 44 patients developing HCC over a median follow-up of 3.5 y	Two phase 3 cohorts: 2156 patients with cirrhosis and 1328 patients with cirrhosis; sub-HR for HCC in medium-risk: 1.77–4.20; high-risk: 6.54–7.51 (reference low risk for both)
Aoki, 2017— VFMAP^46^	Transverse shear wave velocity values, glucose, gender, age, and AFP	1808 patients with chronic liver disease with 49 incident HCCs over a median of 51.6 mo; c-statistic: 0.82 for 5-year HCC risk prediction	358 patients with chronic hepatitis C infection after SVR 3-year HCC incidence: low-risk group—0.96%, high-risk group—18.5%
Gu, 2024— PLEASE algorithm^49^	Liver stiffness measurement, age, gender, etiology of liver disease, and platelet count	1974 patients with F3/4 fibrosis; 5.4% with HCC after a median follow-up of 2.3 y; c-statistic: 0.874	114 patients with chronic liver disease and 183 patients with decompensated cirrhosis; c-statistic: not reported
Semmler, 2022^48^	AFP, alcohol consumption (optional), age, LSM, and albumin	475 patients with F3/4 fibrosis with chronic hepatitis C after SVR and compensated liver disease; 4.6% incidence at a median follow-up of 41 months; c-statistic: 0.893	1500 patients with ACLD after SVR; 4-year HCC incidence: low risk: 3.3%; high risk: 17.6%; c-statistic: not reported

Abbreviations: ACLD, advanced chronic liver disease; AFP, alpha-fetoprotein; HCC, hepatocellular carcinoma; HCV, hepatitis C virus; HR, hazard ratio; LSM, liver stiffness measurement; PLEASE, PLatelet, Elastography, Age, Sex, and Etiologies; SVR, sustained virological response.

## LIMITATIONS OF CURRENT MODELS

There are several notable limitations to existing risk stratification models that limit their clinical validity and ability for widespread implementation. First, many published models do not adequately reflect contemporary etiologies of cirrhosis.^51^ A recent systematic review of prediction models found that patients with ALD and MASLD are poorly represented in both the derivation and validation cohorts.^52^ Given the lower overall incidence of HCC in metabolic liver disease, discrimination may be reduced compared with viral etiologies, potentially limiting clinical utility in these increasingly prevalent populations. Second, existing models largely do not account for competing risks, such as death or transplantation, which may decrease the benefits of surveillance in higher-risk groups, with notable exceptions being the ALICE model and the PAAM score.^37^ Several variables incorporated into HCC risk models—such as platelet count, albumin, and bilirubin—are also strong predictors of mortality in patients with cirrhosis. Furthermore, patients with cirrhosis, particularly with metabolic causes of liver disease, also have significant competing risks from non–liver–related causes of mortality, such as cardiovascular events or extrahepatic cancers, which are not accounted for in existing models, which largely use Cox regression for risk modeling.^53^ Third, while risk-stratified surveillance offers theoretical advantages, these benefits are largely extrapolated from modeling assumptions rather than prospective clinical evidence. Rigorous evaluation in clinical trials is needed to define the real-world risks, benefits, and feasibility of implementing risk-stratified surveillance strategies. Finally, the most well-validated risk stratification markers are clinically derived markers based on variables obtained during routine clinical care, such as demographics and laboratory results. While clinically derived models would allow facile implementation in clinical care and limit health disparities that may occur with stratification that requires specialized testing, the models may lack sufficient power to stratify risk. Future cohort studies in patients with cirrhosis could collect multimodal parameters, including blood tests, imaging, and elastography, to begin to develop richer cohorts for the discovery and validation of multidimensional risk-based surveillance models.^54^

## IMPLEMENTATION OF RISK-BASED SURVEILLANCE

**FIGURE 2:**
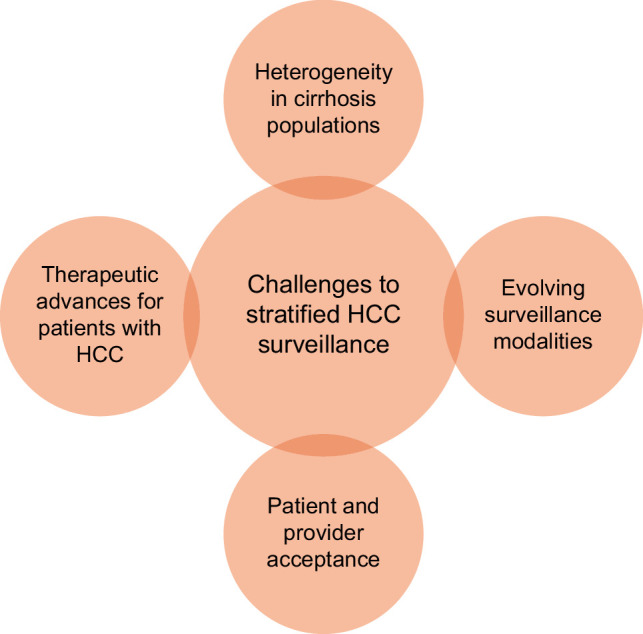
Challenges to risk-stratified HCC surveillance implementation. Abbreviation: HCC, hepatocellular carcinoma.

Implementing risk-stratified surveillance presents several challenges: (1) heterogeneity in cirrhosis populations and risk profiles; (2) provider and patient acceptance; (3) an evolving surveillance modality landscape; and (4) the dynamic relationship between surveillance benefit and therapeutic advances (Figure 2).

1. Populations of patients with cirrhosis can vary significantly by race/ethnicity, socioeconomic status, and etiology of disease. Sufficient validation of the risk stratification schema in mixed populations is necessary to ensure that there is external validity of the models in broad clinical practice settings. The need for specialized testing, such as a biomarker or elastography/imaging, may exacerbate disparities due to access to tests and costs, which may decrease the benefits of a risk-stratified screening paradigm when applied on a population level. In clinical practice, providers typically encounter a variety of disease etiologies in patients with cirrhosis, and thus, disease-specific models for stratification may face significant implementation challenges. Patients frequently have multiple contributing etiologies, limiting the practicality of etiology-based stratification. Disease-agnostic models would increase the practicality of clinical implementation.
2. Provider acceptance of risk-stratified surveillance has been evaluated in a multicenter survey study from the United States, including 305 hepatology providers.^55^ While respondents supported the use of more sensitive modalities (eg, CT or MRI) for high-risk patients, acceptance of surveillance de-escalation in low-risk individuals was limited, with 72.1% continuing surveillance even when the annual incidence was below 0.5%.^55^ Patient perceptions and acceptance of risk-stratified HCC surveillance pathways remain poorly characterized and will be critical to successful implementation.
3. There are several emerging methods being evaluated for HCC surveillance, including novel biomarker-based approaches and imaging-based approaches such as abbreviated MRI.^56^,^57^,^58^ Effective implementation of risk-stratified surveillance will require clearly defined care pathways linking estimated risk to surveillance modality and interval. The integration of novel validated surveillance methods could add complexity to the risk–benefit ratio of stratified surveillance, where modality of surveillance, its cost, and performance characteristics may change the recommended surveillance modality. For instance, a highly sensitive, low-cost, minimally burdensome test could reduce the marginal value of risk stratification.
4. HCC stage at detection strongly predicts outcomes; however, continued advances in systemic therapy and expanding access to curative treatments may alter the relative benefit of early detection across risk strata. These shifting paradigms have been shown in other cancers, with later-stage disease detection showing excellent survival, diminishing the benefits of early detection.^59^ Late-stage HCC remains associated with high mortality; however, over the past decade, several important advances in systemic therapies and increased access to curative therapy, such as liver transplantation, have started to improve outcomes in these high-risk groups.^60^,^61^ The benefits of early detection in the context of improving clinical outcomes deserve attention to understanding the risks and benefits of surveillance overall and in the context of risk-stratified surveillance.

While the implementation of risk-stratified surveillance will invariably face challenges, the potential benefits over current surveillance paradigms are significant. Modeling suggests risk-stratified approaches will result in improved early detection, which will likely result in outcomes for patients with HCC.^62^,^63^ Prospective validation of the scores is necessary as we have little data on real-world implementation of risk-based surveillance in clinical practice and the dynamic nature of risk scores and patient categorization over time. A key practical barrier will be integration into electronic health records, requiring automated data extraction and intuitive clinical decision support tools. Achieving scalable implementation, leveraging artificial intelligence tools, across diverse health systems and electronic health record platforms will be critical to widespread adoption.

## FUTURE OUTLOOK

While there are several current challenges to risk-stratified surveillance validation and implementation, the advent of novel tests for HCC surveillance and their integration into the changing landscape of clinical practice settings will add further complexity. There are emerging data for biomarker-based surveillance with protein and cell-free DNA-based markers that may overcome several of the limitations of current ultrasound-based surveillance.^64^,^65^,^66^ Abbreviated MRI, a limited sequence MRI, is being evaluated head-to-head versus ultrasound in 2 large clinical trials in the United States and France, but has already shown promise in a cohort study.^40^,^67^,^68^ Ideally, risk-stratified surveillance frameworks would be integrated into trials evaluating emerging HCC surveillance modalities. However, such integration requires validated, widely accepted risk stratification schemas before prospective evaluation. Precision surveillance, where the optimal surveillance test is chosen based upon (1) the patient’s underlying risk of HCC and (2) the surveillance modality performance based on patient characteristics (eg, obesity, cirrhosis severity), could further aid in improving the effectiveness of screening programs, albeit with greater complexity.^63^

In conclusion, risk-stratified surveillance offers meaningful advantages over the current one-size-fits-all approach to HCC surveillance. While barriers to validation and implementation remain, more precise balancing of surveillance benefits and harms through risk stratification has the potential to improve HCC-related outcomes.
